# First Study on Nihonium (Nh, Element 113) Chemistry at TASCA

**DOI:** 10.3389/fchem.2021.753738

**Published:** 2021-11-30

**Authors:** A. Yakushev, L. Lens, Ch. E. Düllmann, M. Block, H. Brand, T. Calverley, M. Dasgupta, A. Di Nitto, M. Götz, S. Götz, H. Haba, L. Harkness-Brennan, R-D. Herzberg, F. P. Heßberger, D. Hinde, A. Hübner, E. Jäger, D. Judson, J. Khuyagbaatar, B. Kindler, Y. Komori, J. Konki, J.V. Kratz, J. Krier, N. Kurz, M. Laatiaoui, B. Lommel, Christian Lorenz, M. Maiti, A.K. Mistry, Ch. Mokry, Y. Nagame, P. Papadakis, A. Såmark-Roth, D. Rudolph, J. Runke, L.G. Sarmiento, T.K. Sato, M. Schädel, P. Scharrer, B. Schausten, J. Steiner, P. Thörle-Pospiech, A. Toyoshima, N. Trautmann, J. Uusitalo, A. Ward, M. Wegrzecki, V. Yakusheva

**Affiliations:** ^1^ GSI Helmholtzzentrum für Schwerionenforschung, Darmstadt, Germany; ^2^ Helmholtz-Institut Mainz, Mainz, Germany; ^3^ Johannes Gutenberg-Universität Mainz, Mainz, Germany; ^4^ Department of Physics, University of Liverpool, Liverpool, United Kingdom; ^5^ Department of Nuclear Physics, Australian National University, Canberra, ACT, Australia; ^6^ RIKEN, Wako, Saitama, Japan; ^7^ Department of Physics, University of Jyväskylä, Jyväskylä, Finland; ^8^ Department of Physics, Lund University, Lund, Sweden; ^9^ Indian Institute of Technology Roorkee, Roorkee, India; ^10^ Japan Atomic Energy Agency, Tokai, Japan; ^11^ Łukasiewicz-Instytut Mikroelektroniki I Fotoniki, Warsaw, Poland

**Keywords:** superheavy elements, nihonium, element 113, gas phase chromatography, physical preseparation, TASCA

## Abstract

Nihonium (Nh, element 113) and flerovium (Fl, element 114) are the first superheavy elements in which the *7p* shell is occupied. High volatility and inertness were predicted for Fl due to the strong relativistic stabilization of the closed *7p*_*1/2*_ sub-shell, which originates from a large spin-orbit splitting between the *7p*_*1/2*_ and *7p*_*3/2*_ orbitals. One unpaired electron in the outermost *7p*_*1/2*_ sub-shell in Nh is expected to give rise to a higher chemical reactivity. Theoretical predictions of Nh reactivity are discussed, along with results of the first experimental attempts to study Nh chemistry in the gas phase. The experimental observations verify a higher chemical reactivity of Nh atoms compared to its neighbor Fl and call for the development of advanced setups. First tests of a newly developed detection device miniCOMPACT with highly reactive Fr isotopes assure that effective chemical studies of Nh are within reach.

## 1 Introduction

Relativistic effects strongly influence the chemical properties of superheavy elements (SHE) with atomic number *Z* ≥ 104 (Pyykkö, 1978; Pershina, 2019). General properties of these elements, such as volatility and chemical reactivity, may thus significantly differ from those of their nearest lighter homologues (Schwerdtfeger et al., 2020). As SHE have not been found in nature so far, they have to be synthesized artificially in nuclear fusion reactions at suitable particle accelerators. The production rates of man-made superheavy elements decrease rapidly with increasing *Z*, reaching single atoms per day or less. The nuclear lifetimes drop to seconds or less (Oganessian and Utyonkov, 2015). Chemical studies on SHE are challenging, due to both minimal production rates and the short half-lives of even the most long-lived known isotopes (Türler et al., 2015). These experiments are always preceded by single-atom-at-the-time experiments of their lighter homologues, that can be produced at high rates. Further guidance comes from relativistic quantum chemical predictions of the properties of SHE and their compounds. The most important production method for the creation of SHE is complete fusion followed by sequential evaporation of one or more neutrons. Reactions using intense ^48^Ca ion beams bombarding heavy actinide targets have been used for the discovery of the heaviest elements up to oganesson, element 118 (Oganessian and Utyonkov, 2015).

Increased chemical inertness and corresponding high volatility were predicted for superheavy elements Cn (element 112) and Fl (element 114) due to a large relativistic stabilization of the outermost spherical orbitals *7s* ^*2*^ and *7p*_*1/2*_^*2*^, respectively (Schwerdtfeger and Seth, 2002; Pershina et al., 2008; Hermann et al., 2010; Pershina, 2018). Located between Cn and Fl, Nh (element 113) has one unpaired electron in the outer electron shell with the configuration *7s* ^*2*^ *7p*_*1/2*_^*1*^. The unpaired *p*_*1/*2_-electron may cause increased chemical reactivity, while the spherical *7p*_*1/2*_ orbital is relativistically stabilized. Accordingly, Nh is predicted to be more reactive upon adsorption on gold and quartz surfaces than its neighbors Cn and Fl, but less reactive than Tl, its nearest homologue in group 13 (Pershina et al., 2009; Fox-Beyer and van Wüllen, 2012; Rusakov et al., 2013; Pershina, 2016; Pershina et al., 2021). The large spin-orbit splitting in the *7p* shell is reflected in its chemical behaviour - Nh appears to be more inert than lighter homologues in the group 13, but can form stable compounds, such as hydrides or hydroxide NhOH. A bulk property of Nh, its cohesive energy was estimated from calculations of the adsorption energy on gold to be about -0.7 eV (Trombach et al., 2019). Adsorption energies of gaseous hydroxides MOH (M = In, Tl, and Nh) on gold were calculated using molecular and periodic relativistic DFT calculations The strongest binding to gold has Nh hydroxide, NhOH, which is caused by increasing molecular dipole moments and decreasing stability of the hydroxides in group 13 InOH > TlOH > NhOH (Pershina and Ilias, 2019).

The most effective experimental approach in contemporary SHE chemistry research is the gas-solid chromatography method (Zvára, 2008; Türler and Pershina, 2013). The study of chemical processes and the detection of single SHE atoms via registration of their nuclear decay take place in a chromatography column adapted to fulfil both tasks. Thus, many theoretical investigations on atomic and chemical properties of the heaviest elements performed during the last 2 decades focused on the interaction between single atoms of SHE and solid surfaces employed in the experiments, such as SiO_2_ and Au. Relying on the expected high volatility and weak chemical reactivity, the gas-solid chromatography method was used for Cn and Fl adsorption studies, mainly on Au surface (Türler et al., 2015). In accordance with theoretical predictions, weak bonding to Au was experimentally confirmed for both elements, which remained in the elemental state under the experimental conditions (Eichler et al., 2007; Eichler et al., 2010; Yakushev et al., 2014; Yakushev and Eichler, 2016). A chemical inertness towards polytetrafluoroethylene (PTFE) and SiO_2_ surfaces allowed for effective transport of Cn and Fl to the gas chromatography and detection setup by an inert-gas flow. In this setup, the interaction with a Au surface was studied, resulting in the observation of a relatively weak metal-Au interaction, thus confirming the predicted relativistic stabilization of the *7s* and *7p*_*1/2*_ (sub)shells in Cn and Fl (Eichler et al., 2007; Eichler et al., 2010; Yakushev et al., 2014; Yakushev and Eichler, 2016).

Nh isotopes for chemistry studies can be produced as decay products following nuclear fusion reactions between ^48^Ca and ^243^Am or ^249^Bk targets resulting in the production ofisotopes of Mc (element 115) and Ts (element 117) isotopes, respectively (Oganessian and Utyonkov, 2015). The nuclear reaction ^243^Am (^48^Ca,3n)^288^Mc has a higher cross section of about 10 pb (Oganessian et al., 2013; Rudolph et al., 2013; Rudolph et al., 2014; Gates et al., 2015; Rudolph et al., 2015; Forsberg et al., 2016). The second member of the decay chain, ^284^Nh, is accessible for chemical studies after an *α*-decay of the short-lived mother nuclide ^288^Mc. However, the same experimental approach for adsorption studies with Nh is expected to be more challenging due to the higher reactivity of Nh atoms with any surface. Furthermore, theoretical predictions indicate that Nh might readily form compounds in the gas phase, e.g., NhOH, bringing ambiguity to the question about which chemical species will be present in the adsorption studies (Pershina et al., 2009). Following an assessment of previous chemical studies of Nh at the Flerov Laboratory of Nuclear Reactions (FLNR) in Dubna (Russia), we present the results of the first adsorption study of Nh on SiO_2_ and Au surfaces, performed behind the gas-filled separator TransActinide Separator and Chemistry Apparatus (TASCA) at the GSI Helmholtzzentrum für Schwerionenforschung (GSI) in Darmstadt, Germany. The results of all previous studies on Nh chemistry call for further optimization of the existing techniques to facilitate future gas-chromatography experiments on Nh and, possibly, Mc.

## 2 Initial Attempts at Chemical Studies of Nh at the FLNR

**FIGURE 1:**
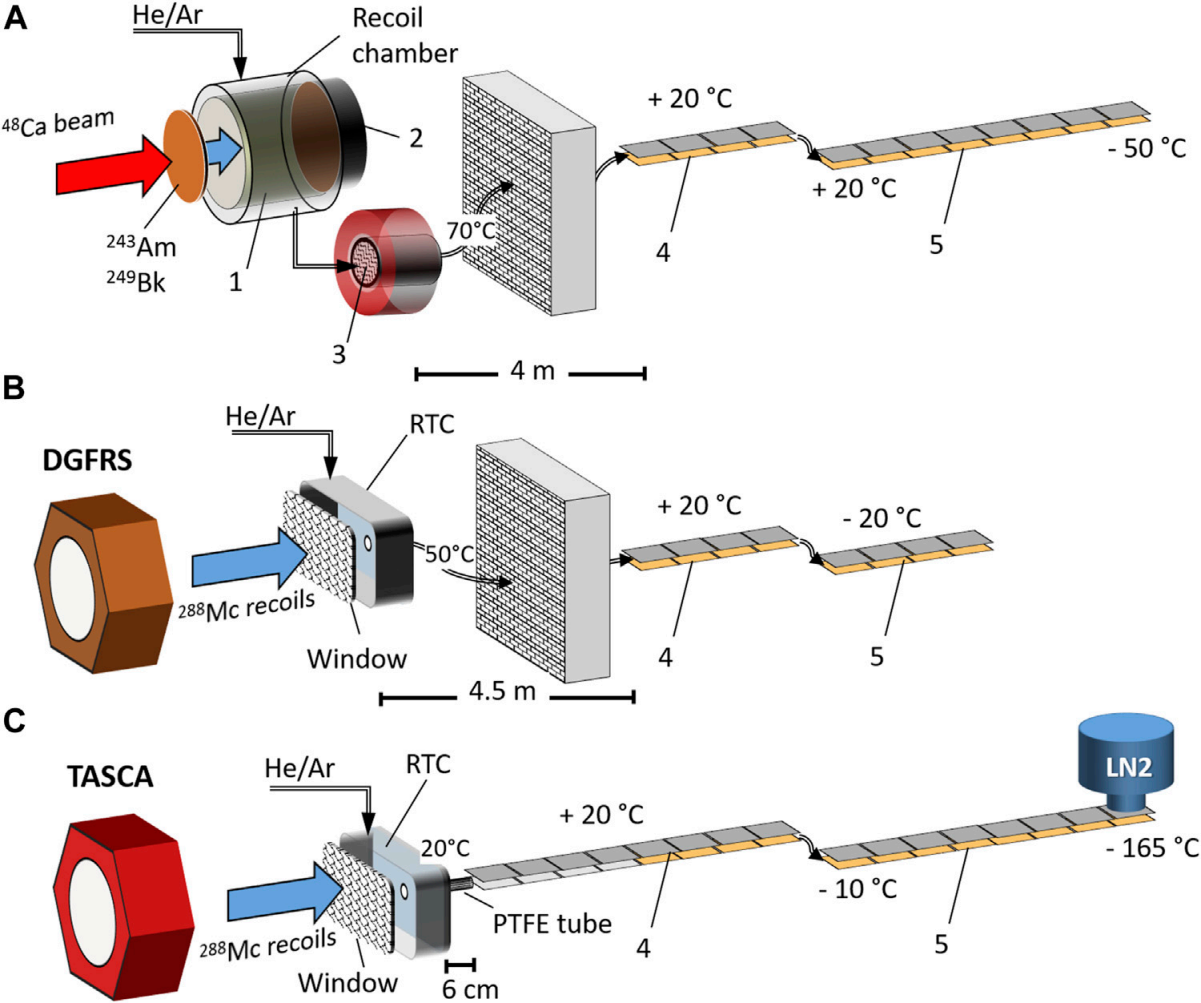
Experimental setups used in the Nh chemistry experiments: **(A)**–setup used in the pioneering experiments at the FLNR without pre-separation (Dmitriev et al., 2014; Türler et al., 2015); **(B)**–setup used in the FLNR experiment behind the DGFRS (Aksenov et al., 2017); **(C)**–setup used at the GSI behind the TASCA separator (this work).

In the past, a series of chemical experiments with Nh were conducted by a collaboration led by FLNR (Dmitriev et al., 2014; Türler et al., 2015; Aksenov et al., 2017). In total, five experiments were performed using a setup as depicted in Figure 1A. Stationary ^243^Am or ^249^Bk targets were irradiated with ^48^Ca beams. Because of the limited availability of the ^249^Bk target material, most experiments were performed by irradiation of ^243^Am targets. The main product of those irradiations was ^288^Mc, produced in the 3n-evaporation channel following the complete fusion reaction. The SHEs of interest were thermalized in a He/Ar gas mixture directly behind the target and transported by the gas flow to a detection setup. The transport line from the chamber, where the ions recoiling from the target were thermalized, to a detection setup was 4 m or longer in all experiments performed at FLNR. Since the half-life of ^288^Mc (*T*_*1/2*_ ≈ 170 ms) is too short for the applied technique, the chemical experiments were focused on adsorption studies of its longer-lived *α*-decay product, ^284^Nh (*T*_*1/2*_ ≈ 1 s), on a Au surface. The Au-covered PIN photodiodes formed a detection channel, consisting of one or two detector arrays kept either at constant ambient or lower temperatures, or with a negative longitudinal temperature gradient. In all these experiments without pre-separation, an insert made of quartz was installed in the recoil chamber to prevent collisions of thermalized products with the metallic walls. In addition, a hot quartz-wool filter was installed to avoid the transport of non-volatile species by aerosol particles, which can be formed by the intense beam interacting with the recoil chamber back wall.

The products from the fusion-evaporation reaction between the ^48^Ca ion beam and ^243^Am (^249^Bk) recoiling from the target, i.e., primarily ^288^Mc (^294^Ts), were thermalized inside a recoil chamber (1) placed directly behind the target or in a Recoil Transfer Chamber (RTC) at the focal plane of the Dubna Gas-Filled Recoil Separator (DGFRS) or of TASCA. Quartz was inserted inside the recoil chamber in the experiments without pre-separation (A), where the beam traversed through the gas before it was stopped in a beam stop (2). To prevent the break-through of aerosol particles, a quartz wool plug heated to 600°C (3) was installed at the exit of the recoil chamber. In experiments behind a preseparator, ^288^Mc ions penetrated a window separating the RTC volume from the separator volume. Two detector arrays, (4) and (5), placed in a row were used in the experiments (see Figure 1): A) a Au-covered detector array kept at ambient temperature (4) and a Au-covered detector array with a negative temperature gradient from +20°C to −50°C (5) as used in (Dmitriev et al., 2014); B) a Au-covered detector array kept at ambient temperature (4) and a Au-covered detector array kept at −20°C (5) as used in (Aksenov et al., 2017); C) two COMPACT detector arrays were used in the present study: a combined detector array (4) consisted of 16 SiO_2_-covered detector pairs and 16 Au-covered detector pairs, kept at ambient temperature, and a Au-covered detector array with a negative temperature gradient from −10°C to −165°C (5). The thickness of the SiO_2_ and Au layers on the detector surface was 30–50 nm. The recoil chamber (or RTC) was connected to the detection setup by PTFE capillaries of different lengths kept at different temperatures, as is indicated in Figure 1.

No events which could be assigned to ^284^Nh were found in the first three runs at Dubna using ^243^Am targets although observation of 10–20 decay chains from ^284^Nh had been expected based on the known efficiency and a total beam integral of 1.35.10^19^ particles (Türler et al., 2015). One decay chain was observed in the irradiation of a ^249^Bk target with ^48^Ca ions for an applied beam dose of 9·10^18^. This event was tentatively assigned to ^286^Nh (Türler et al., 2015). Five decay chains were reported from the experiment performed at FLNR in 2013, where again a ^243^Am target was irradiated with 8·10^18 48^Ca ions. The registered decay chains were attributed to ^284^Nh (Dmitriev et al., 2014), but no assignment of a chemical form was given. As discussed already in (Türler et al., 2015), the attribution to ^284^Nh can be considered somewhat ambiguous because in the mismatch of nuclear properties of some decay-chain members as compared with high-resolution and high-statistics data. About one hundred long decay chains from ^288^Mc consisting of five *α*-decays and spontaneous fission have been registered in focal-plane measurements at the recoil separators DGFRS (Oganessian et al., 2013; Oganessian and Utyonkov, 2015), TASCA (Rudolph et al., 2013), and BGS (LNBL, Berkeley, United States) (Gates et al., 2015). Thus, the *α*-particle energies and the half-lives along the decay chain starting with ^288^Mc are well-known (Forsberg et al., 2016). The decay properties of the decay chain members reported from the Nh chemistry study at FLNR (Dmitriev et al., 2014) disagree with the known properties of decay-chain members. The registered *α*-decay energies of the two decay chain members, ^280^Rg and ^276^Mt, are significantly lower, and their lifetimes are significantly longer. In particular, the mean lifetime of the decay chain member ^276^Mt, determined in the first chemical study at FLNR based on three events (Dmitriev et al., 2014), is 40 times longer than the known value (*T*_*1/2*_ = $0.69_{-0.07}^{+0.09}$ *s*) (Forsberg et al., 2016).

Due to a rather high background in the *α*-decay spectra from volatile byproducts of multi-nucleon transfer reactions, the data quality in the experiments without pre-separation hampered the safe identification of Nh and its daughters (Türler et al., 2015; Dmitriev et al., 2014). This is a particular problem in SHE chemistry experiments with volatile species, in which no physical pre-separation from unwanted volatile byproducts; e.g., Rn isotopes, and their progenies, is employed. Such a background, however, can efficiently suppressed with an electromagnetic recoil separator (Düllmann et al., 2005; Wittwer et al., 2010). By using a recoil separator with a finite transmission for guiding evaporation residues to the RTC window, the overall efficiency of a chemistry experiment reduces, however, allows the detection of rare events under low-background conditions. To overcome this limitation, the most recent experiment on Nh chemistry was conducted behind the DGFRS, which was used as a pre-separator (Figure 1B) (Aksenov et al., 2017). The observation of four decay chains from ^284^Nh was expected based on known production rates. However, not a single one was observed (Aksenov et al., 2017). This non-observation was explained by losses due to the adsorption of Nh atoms on the PTFE surfaces of the 4.5-m long transport line. A rather high adsorption enthalpy limit for the adsorption of Nh on a PTFE surface, *−ΔH*_*ads*_(*Nh*) > 45 kJ/mol was concluded based on the non-observation (Aksenov et al., 2017). As an explanation for the observation of five decay chains in the experiment reported by (Dmitriev et al., 2014), the possible formation of a volatile Nh compound, presumably NhOH, was suggested (Aksenov et al., 2017). This result implies that none of the previous experiments without pre-separation performed at FLNR could have registered Nh in the elemental form.

## 3 Experiments on Nh Chemistry at GSI

### 3.1 Preparatory Experiments With Homologues Hg, Tl, and Pb

Prior to the Nh chemistry experiment at GSI, adsorption studies of the non-volatile metals Tl and Pb and the volatile metal Hg were performed behind TASCA with a setup similar to that shown in Figure 1C, using a SiO_2_-covered and a Au-covered detector array (Lens et al., 2018). Short-lived Tl and Pb isotopes were produced in nuclear fusion reactions with ^50^Ti or ^48^Ca ion beams bombarding ^141^Pr and ^144^Sm targets, respectively. Reaction products were separated in TASCA, and thermalized inside the RTC. The diffusion-controlled deposition of both Tl and Pb was observed in the first SiO_2_ detector channel, whereas Hg passed the first detector array and adsorbed on Au (Lens et al., 2018). A diffusion-controlled deposition was observed in both detector arrays: for Pb and Tl isotopes on the SiO_2_ detector surface and for Hg isotopes on the Au detector surface. A lower limit of the adsorption enthalpy of *−ΔH*_*ads*_ ≥ 67 kJ/mol (Lens et al., 2018) was determined for Tl and Pb on a SiO_2_ surface and for Hg on a Au surface through Monte Carlo (MC) simulations using the mobile adsorption mechanism (Zvára, 1985). A transport efficiency from the RTC into the Cryo Online Multidetector for Physics And Chemistry of Transactinides (COMPACT) of 25% was obtained for Pb isotopes, while 62% was found for the less reactive Hg (Lens et al., 2018). This difference points at larger adsorption losses inside the RTC and the connecting tube for Pb compared to Hg. Due to the small *α*-decay branches of the produced Tl isotopes, no quantitative evaluation of the chemical yield for Tl was possible. However, the chemical yield estimated for Tl should not exceed that of Pb (Lens et al., 2018).

**FIGURE 2:**
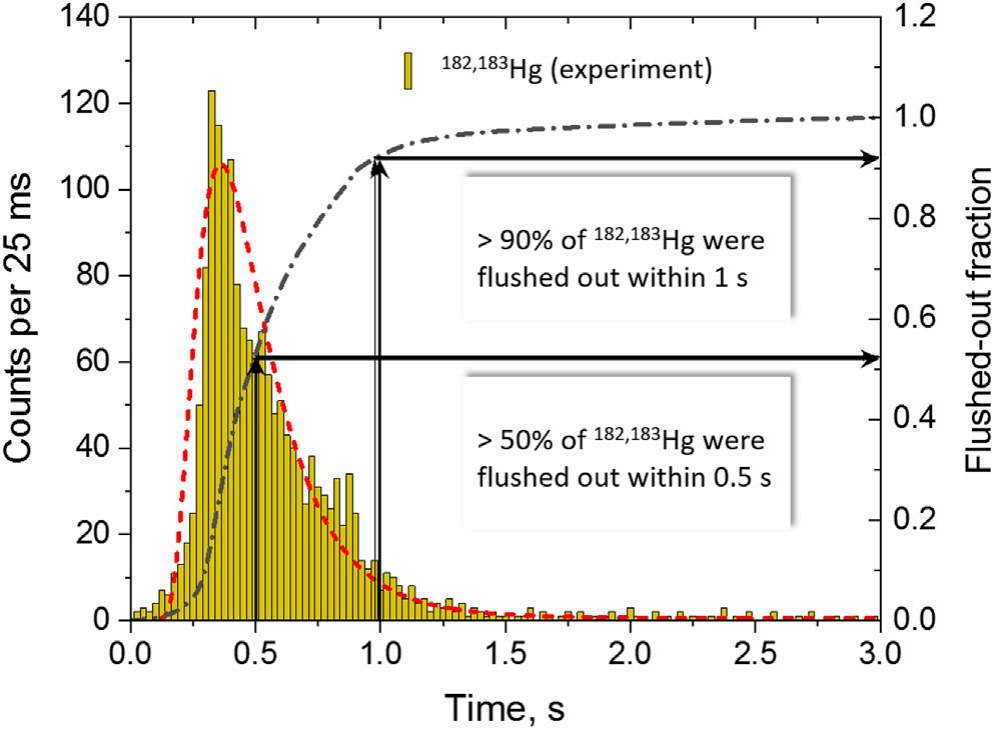
Time distribution for the time difference between the start of 0.1 s-long irradiations and the time of decay-in-flight, measured for ^182,183^Hg in the first COMPACT array (yellow bars). The red dashed line is a lognormal-distribution fit function with the peak position at 0.35 s. The black dashed-dotted line represents the time-dependent function for the integrated experimental flushed-out fraction of ^182,183^Hg detected in the first COMPACT array within a 3 s period after the start of irradiation.

Besides losses due to irreversible adsorption before reaching the detectors, the rate of observed atoms may be reduced due to nuclear decay during transport. These losses depend critically on the time required for transporting the atoms from the production site to the detector; in our case, the COMPACT arrays. Transit times through TASCA are on the order of 1 μs and are negligible compared to the time needed for flushing out the atoms from the RTC to COMPACT. The transport time to the detection setup was measured with short-lived *α*-decaying ^182,183^Hg isotopes using a pulsed beam sequence (0.1 s beam on, 5 s beam off). The time elapsing between the start of each beam pulse and the observed decay-in-flight of Hg isotopes over the first 16 SiO_2_-covered detector pairs was registered. This measurement allowed determination of the flush-out time distribution. The measured flush-out time distribution and the integrated fraction of flushed-out ^182,183^Hg atoms as a function of time are depicted in Figure 2, together with a lognormal distribution fit with the peak position at 0.35 s. The measured distribution reveals that 50% of Hg atoms were extracted from the RTC and were transferred into the first COMPACT array within 0.5 s. About 90% of all Hg atoms were extracted within 1 s. Accordingly based on the extraction efficiency for Hg, the losses of ^284^Nh (*T*_*1/2*_ ≈ 1 s) due to decay during their transfer from the RTC volume into COMPACT were estimated not to exceed 50%.

The measurement of decay-in-flight of Hg isotopes in the first detector array with time and position resolution enabled us to determine the retention time of Hg in the SiO_2_ chromatography channel. The travelling time of the weakly-interacting Hg along the 16 cm-long SiO_2_-covered detector array was estimated to be 50 ms at ambient temperature.

### 3.2 The First Chemical Study of Nh at TASCA

Building upon successful Fl chemistry experiments at the gas-filled separator TASCA (Yakushev et al., 2014; Yakushev and Eichler, 2016), the gas-solid interaction study of Nh atoms with SiO_2_ and Au surfaces was next targeted. The first attempt at adsorption studies of Nh at TASCA was performed in 2016. A pulsed ^48^Ca^+10^ beam (5 ms beam on, 15 ms beam off, 45 Hz repetition rate) from the UNILAC accelerator with a beam energy of 5.47 MeV/amu (before the Ti target substrate) bombarded a ^243^Am target wheel, which rotated synchronously with the pulsed beam (Jäger et al., 2014). Four ^243^Am target segments were deposited on 2.2(1) μm Ti foils by molecular plating (Runke et al., 2014). The average target thickness of the four ^243^Am segments was 0.80(1) mg/cm^2^ of ^243^Am (isotopic enrichment: 99.7%). A beam integral of 4.4(1)·10^18^ was collected during the 20-days long irradiation. TASCA was filled with He at a pressure of *p*_He_ = 0.8 mbar and set to a magnetic rigidity of *B·ρ* = 2.21 Tm to center ^288^Mc recoils in the TASCA focal plane (Khuyagbaatar et al., 2012; Rudolph et al., 2013). The nominal transmission efficiency in TASCA to focus the recoiling ^288^Mc ions into the RTC window area is about 40%. In the present experiment, a malfunction of the power supply of the second quadrupole magnet led to a reduced transmission efficiency of approximately 20%, deduced from independent studies with ^254^No evaporation residues produced in the ^48^Ca + ^208^Pb reaction. Nevertheless, under these conditions eleven ^288^Mc recoils were expected to pass through the RTC entrance window (60 × 40 mm^2^), made of 3.6 μm Mylar™ film on a stainless-steel supporting grid with 80% transparency. Recoiling EVRs were then thermalized in a He/Ar (1/1) gas mixture at 1 bar inside the 48-cm^3^ large RTC volume and were flushed out to COMPACT detector arrays at a gas flow rate of 2 L/min. The inner RTC wall was covered with a Teflon™ layer. A 6-cm long PTFE tube (i.d. 4 mm) connected the RTC volume to the detection setup, so that atoms thermalized inside the RTC encountered only non-metallic surfaces before they entered COMPACT.

A gas chromatography setup similar to that used in Fl studies (Yakushev et al., 2014) was applied. Whereas the first COMPACT array was kept at room temperature, a negative temperature gradient from −10°C to −165°C was applied along with the second one (Figure 1C). The first COMPACT array was modified to have two different detector surfaces: the 16 first individual detector pairs were covered with SiO_2_ followed by 16 Au-covered detector pairs. The second COMPACT array consisted of 32 Au-covered detector pairs (Figure 1C).

## 4 Results and Discussion

### 4.1 Results of the Nh Experiment at TASCA

In this experiment, the observation of four long decay chains originating from ^284^Nh was expected given that the chemical behaviour of Nh is similar to that of Fl. However, no time- and position-correlated decay chains, consisting of one or several *α* decay(s) terminated by a spontaneous fission (SF) event, were registered. This indicates a reactivity of Nh that is higher than that of Fl on a confidence level of >95% for small numbers. Only one SF event consisting of two coincident fission fragments but without *α*-decay precursors was detected in the fifth detector pair of the first COMPACT array, on the SiO_2_ surface. The probability for the detection of a single *α* decay is about 80%. Thus, the probability that the SF event originated from the long decay chain beginning in ^288^Mc or ^284^Nh is almost negligible. However, it could originate from shorter decay chains, which end by the spontaneous fission of Nh or Rg isotopes; according to the nuclear decay properties given in (Oganessian et al., 2013; Rudolph et al., 2013; Rudolph et al., 2014; Gates et al., 2015; Oganessian and Utyonkov, 2015; Rudolph et al., 2015; Forsberg et al., 2016).

There is a non-zero probability that the non-observation of Nh decay chains is due to low statistics. However, it may also be caused by an irreversible adsorption of Nh inside the RTC or on the inner wall of the PTFE tube connecting the first COMPACT array to the RTC. We applied MC simulations for the case of mobile adsorption (Zvára, 1985) to estimate possible losses of ^284^Nh due to retention in the connecting tube. The results of these simulations suggest that the interaction strength has to be rather high in order to explain significant losses of ^284^Nh atoms in the connecting tube: more than 95% of ^284^Nh atoms would decay in the connecting tube if the adsorption enthalpy value of Nh atoms on the PTFE surface is at least 50 kJ/mol. Such high values for the adsorption enthalpy of Nh on the PTFE surface are indicative of chemisorption processes. This agrees with the non-observation of Nh events in the experiment behind the DGFRS, where a limit for the adsorption enthalpy on PTFE of *−ΔH*_*ads*_ > 45 kJ/mol (95% c. l.) was determined (Aksenov et al., 2017). Another possible explanation for the non-observation of Nh in the detector array is the formation of a less-volatile chemical compound of Nh, e.g., during the thermalization process inside the RTC. The main gas impurities (O_2_ and H_2_O) were kept at a level of a few ppm, yet gas-phase reactions between Nh atoms and gas impurities cannot be completely excluded. In that case, the formation of NhOH could be expected, which is predicted to be non-volatile (Pershina et al., 2009).

### 4.2 Potential Chemical Reactions of Mc and Nh Inside the RTC

^284^Nh is produced via *α*-particle emission from the nuclear reaction product ^288^Mc that is thermalized in the He/Ar gas inside the RTC (Figure 1C). Thus, the reactivity of both Mc and Nh has to be taken into account. Nihonium and moscovium are predicted to be less reactive than their nearest homologues Tl and Bi, respectively (Pershina et al., 2009; Fox-Beyer and van Wüllen, 2012; Rusakov et al., 2013; Pershina, 2016; Pershina and Iliaš, 2019; Trombach et al., 2019; Pershina et al., 2021). The calculated first ionization potential (*IP*) of Nh (7.306 eV) (Eliav et al., 1996) is larger than that of Tl (6.11 eV). However, it is similar to that of Pb (7.42 eV) and significantly smaller than that of Hg (10.39 eV). Moscovium is predicted to be chemically even more reactive than nihonium with *IP*(Mc) = 5.574 eV (Borschevsky et al., 2015).

The inner walls of the RTC and the entrance into the COMPACT array were covered with PTFE. Thus, Mc and Nh atoms encountered only PTFE surfaces during the transport to the first SiO_2_-covered detector channel. PTFE is known as a very inert hydrophobic material, and the adhesion of different chemical species on the PTFE surface is very low (Renfrew and Lewis, 1946). However, the PTFE surface can be chemically modified by etching with alkali metals. Alkali metals, e.g., Li, react with the fluorine atoms of the PTFE surface, forming fluorides (Jansta et al., 1975). The amount of adsorbed volatile contaminants and other impurities was reduced by purifying the gas to the level of a few ppm by circulating it in a loop, in which purifying cartridges MonoTorr and MicroTorr (SAES) were installed.

Taking into account the *IP* values for Nh and Mc, one can expect a larger chemical reactivity with the surface and gas impurities leading to a reduced transport yield of both elements from the RTC to COMPACT compared to that of Hg.

### 4.3 Chemical Reactivity of Nh and Its Homologue Tl With a SiO_2_ Surface

The nearest lighter homologue of Nh in group 13 is Tl. The growing spin-orbit energy splitting along the group reaches 4.67 eV in the *6p* orbital (Eliav et al., 2016). Thus, the dominant oxidation state of Tl is +1, in contrast to +3 of the lighter group members. The formation of TlOH is expected through the interaction of Tl atoms with a quartz surface. Indeed, gas-chromatography studies of Tl adsorption on quartz in different gases and in vacuum point to the formation of TlOH, which is not volatile below about 300°C (Serov et al., 2013; Steinegger et al., 2016). Despite a predicted lower reactivity of atomic Nh compared to Tl, the formation of NhOH upon contact of a Nh atom with a hot SiO_2_ surface of the quartz wool plug is highly likely, as also suggested by the authors of the second Nh chemistry study at FLNR (Aksenov et al., 2017). Theoretical predictions of NhOH properties compared to TlOH suggest that NhOH molecules maybe even more strongly adsorbed from the gas phase than TlOH due to their larger dipole moment and anisotropic polarizability (Pershina and Ilias, 2018). Thus, the transport losses of NhOH in a PTFE capillary may be comparable to that of Nh in the elemental state or even higher.

### 4.4 Development of a New miniCOMPACT Detector

**FIGURE 3:**
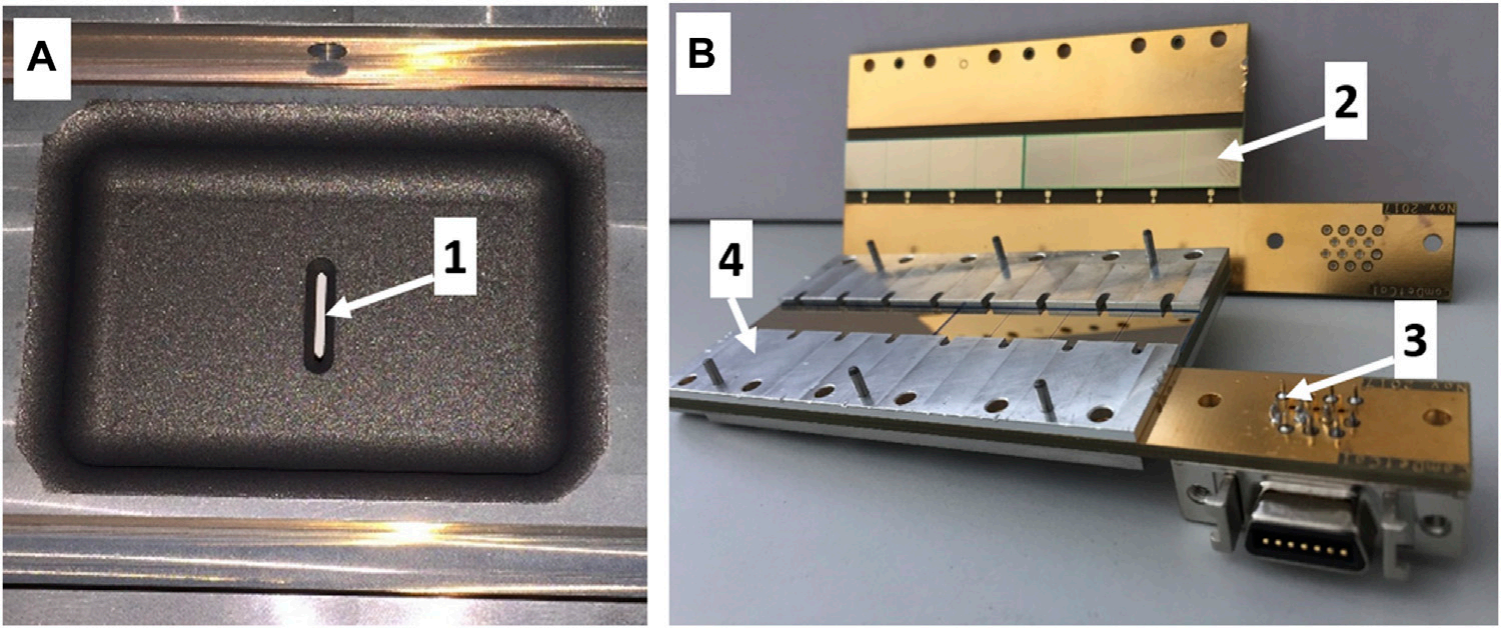
Pictures of the RTC-miniCOMPACT setup showing the RTC **(A)** and the open Au-covered miniCOMPACT array **(B)**. The inner volume of the RTC is 40 × 60 × 20 mm^3^; the exit slit (1) is 10 × 1 mm^2^. One half of the detector channel is made up of eight pairs of silicon PIN diodes (2) (10 × 10 mm^2^) mounted on a PCB (3). The spacer (4) keeps the distance of 1.6 mm between top and bottom detectors in the closed array.

The non-observation of Nh atoms in the experiments with background suppression at two different laboratories (Aksenov et al., 2017; this work) points at possible losses of Nh atoms during the transfer from the RTC to the detection setup. A significant gain in the transport efficiency of reactive or non-volatile species is required, calling for the development of a new setup, which allows a fast and efficient transport to a chromatography and detection channel. This triggered the development of a detection device that can be attached directly to the exit of the RTC, avoiding any connecting tube. Reactive species are immobilized in a gas chromatography channel (Türler and Pershina, 2013) upon their first contact, resulting in an exponentially decreasing deposition within the first few centimeters. Based on the sum of all previous Nh chemistry experiments, a new, short “miniCOMPACT” array was constructed and mounted directly on the back-plate of the RTC. An 8 cm-long miniCOMPACT consists of eight pairs of the same PIN diodes as used in regular COMPACT arrays (Yakushev et al., 2014; Lens et al., 2018) mounted on a printed circuit board (PCB), as depicted in Figure 3. Two such detector arrays on PCBs form a gas-chromatography channel. Two versions of the miniCOMPACT detector were manufactured, one with a Au surface and a second one with a SiO_2_ surface. The miniCOMPACT unit can be attached to a RTC (Figure 3A) such that the gap between the gas volume and the first detector pair is only 1 mm. Alternatively, it can be used with a buffer-gas stopping cell fitted with electrical fields to allow for a even faster flush-out of thermalized recoil ions (Götz et al., 2021). These units were tested in an online experiment at TASCA. The Au-covered miniCOMPACT detector (Figure 3B) was utilised for direct comparative measurements of the transport efficiencies from the RTC to miniCOMPACT for short-lived Hg and Fr isotopes. The SiO_2_-covered miniCOMPACT detector was used in a separate measurement at TASCA to detect Fr isotopes extracted from the buffer-gas stopping cell (Götz et al., 2021).

The transport efficiency from the RTC into the miniCOMPACT was measured with short-lived ^178-180^Hg and ^202-205^Fr produced in the fusion-evaporation reactions with a 5.9 MeV/u^40^Ar^+9^ beam irradiating ^144^Sm and ^169^Tm targets, respectively. The targets were prepared by evaporation of Sm_2_O_3_ and metallic Tm on 2.2(1) μm Ti foils and had thicknesses of about 0.5 mg/cm^2^. The inner volume of the RTC was separated from the TASCA volume by a 6-μm thick Mylar^®^ window. The reaction products recoiling from the target were physically separated using TASCA, and thermalized in a He/Ar (1/1) gas mixture inside the RTC. The measured flush-out efficiency of the reactive alkali metal Fr was compared to that of the volatile and relatively inert metal Hg. While the efficiency for Hg isotopes was expectedly higher (∼58%), a substantial efficiency for Fr isotopes (∼30%) was determined. The distributions along the detector channel were similar for both elements following the diffusion-controlled deposition on the Au surface. The lower transport efficiency of Fr can be explained by its higher chemical reactivity, resulting in larger adsorption losses on the inner walls of the RTC and in the connecting slit.

## 5 Conclusion

Elucidating the adsorption behaviour of Nh on different surfaces is currently one of the hottest topics in the field of superheavy element chemistry. Nh has one unpaired electron in the valence shell and is, therefore, expected to be chemically reactive. To date, several attempts to study the interaction of Nh with Au were reported from FLNR, Dubna. The chemical results of the first studies without physical pre-separation (Dmitriev et al., 2014; Türler et al., 2015) suffered from substantial background in the nuclear-decay spectra and from the unconvincing reliability of spectra and ambiguity in the assignment of decay chains to ^284^Nh. The non-observation of decay chains from ^284^Nh in the experiment behind the DGFRS (Aksenov et al., 2017), and in the present study behind TASCA, implies a higher reactivity of Nh as compared with Cn and Fl, both having (quasi-)closed-shell configurations. The present first chemical Nh study at TASCA resulted in the observation of one SF event, which cannot be clearly assigned to the decay of ^284^Nh or its progenies. These results called for the development of an advanced setup. We therefore developed the new miniCOMPACT detector array, which does not require any transport line between the RTC and the detection setup. Initial tests of this approach with highly reactive Fr isotopes gave an encouraging value of 30% for the absolute transport yield suggesting that a chemical study of Nh and Mc is now within experimental reach.

## Data Availability

The raw data supporting the conclusion of this article will be made available by the authors without undue reservation.
